# CBCT for estimation of the cemento-enamel junction and crestal bone of anterior teeth

**DOI:** 10.1515/med-2020-0211

**Published:** 2020-08-03

**Authors:** Agnieszka Srebrzyńska-Witek, Rafał Koszowski, Ingrid Różyło-Kalinowska, Magdalena Piskórz

**Affiliations:** Private Practice, Kossak-Szczuckiej Street 7/1, 40-578, Katowice, Poland; Academic Center of Dentistry and Specialized Medicine, Pl. Akademicki 17, 41-902, Bytom, Poland; Department of Dental and Maxillofacial Radiology, Medical University of Lublin, Poland, Karmelicka Street 7, 20-081, Lublin, Poland

**Keywords:** cone-beam computed tomography, three-dimensional imaging, mandible, alveolar process

## Abstract

The aim of the study is to evaluate the usefulness of cone-beam computed tomography (CBCT) in the assessment of the relationship between the cemento-enamel junction (CEJ) and bone crest of the anterior mandibular cortex. The study population comprised 39 males and 61 females, aged 18–71. A GENDEX GXCB-500 machine, i-CAT Vision and CorelDraw 9 software were used. The distances between the CEJ and bone crest at buccal and lingual sides of six anterior mandibular teeth were measured. Descriptive statistical methods, Student’s *t*-test and ANOVA were used. The mean distance between the bone crest and CEJ was 2.32 mm ± 0.78 mm at the buccal and 2.52 mm ± 0.85 mm at the lingual side. It was found that in males aged over 50 years, the mean distance at the buccal side was 2.84 mm ± 0.79 mm and was significantly higher than in males aged 49 and less – 2.08 mm ± 0.41 mm. The mean distance at the lingual side was 3.28 mm ± 1.08 mm and was significantly lower in the age group of 49 years and less – 2.10 mm ± 0.41 mm. CBCT allows determining the distance between the CEJ and crestal bone margin at buccal and lingual sides. The data provide crucial information for planning orthodontic treatment, implant placement and periodontal therapy.

## 1 Introduction

The alveolar bone structure, especially its vestibular cortex, is crucial for the aesthetics of smile. Its morphology influences the state of gingiva, and in the case of tooth loss, to a great extent it decides on the possibility of reconstruction of missing teeth, comparable to the natural dentition. The volume of bone covering the vestibular surfaces of teeth can be diminished both in the horizontal and vertical planes due to the influence of numerous local and systemic factors [7,10].

Horizontal and vertical dimensions of the maxillary and mandibular alveolar bone are strictly interrelated. A thin vestibular bone contributes to a decrease in cortical bone height, thus leading to dehiscences and fenestrations. Dehiscence is a defect in the vestibular or lingual cortex causing exposure of the dental root towards its apex deeper than 4 mm in relation to the bone level at mesial and distal surfaces of any given tooth. On the other hand, fenestration is a limited defect of the cortical bone exposing the underlying root surface, but it is not connected with the alveolar cortex margin. Fenestrations and dehiscences affect most commonly the anterior maxilla and mandible [19]. Usually such defects are located at the periapical or medial part of the dental root, and this phenomenon is common. Most of the researchers state that the prevalence of dehiscences falls in the range of 0.99–13.4%, while that of fenestration between 0.23% and 13.9% [7,19,23]. However, some authors claim that this phenomenon is even more prevalent. Nimigean et al. [19] examined 138 skulls of Caucasian individuals and found fenestrations in about 70% of cases, mostly in the maxilla, while dehiscences in about 54%, more commonly in the mandible. Rupprecht et al. [23] discovered fenestrations in 61.6% of 146 skulls of contemporary USA citizens (both Caucasian and Afroamerican), and dehiscences in 40.4% of cases.

Particular anatomical conditions are responsible for the increased prevalence of gingival recessions in anterior maxillary and mandibular teeth. The decrease of the attachment level leads to exposure of the dental cervix and root. This pathology is a complex lesion due to multifactorial aetiology. It is believed that anatomical conditions such as height and width of the outer alveolar cortex as well as the tooth topography are vital. Another group of factors includes the so-called trigger factors, and their influence is especially marked in the presence of unfavourable anatomical conditions. Therefore, gingival recessions occur more frequently in individuals in whom the maxillary and mandibular vestibular cortex covering the teeth is thin and the cancellous bone is absent. Such a situation results in compromised vascularisation of this bone fragment, thus hampering the supply of nutrients and accelerating its loss when contributing factors occur [7,10].

Cone-beam computed tomography (CBCT) allows precise measurements of the maxillary and mandibular alveolar bone. However, so far the evaluations of the bone morphology in relation to dental treatment planning have been conducted on anterior maxilla. Much less research was focused on the anterior mandible despite particular anatomical conditions in this area as described above. Therefore, the aim of this study is to assess the usefulness of CBCT imaging in determination of the relationship between the cemento-enamel junction (CEJ) and the crestal bone margin at buccal and lingual mandibular alveolar processes taking into consideration the utility of such information in clinical applications.

## 2 Methods

The material comprised 100 CBCT scans taken in the Radiological Lab of the Jomadent Health Center in Dąbrowa Górnicza (Poland) in the years 2010–2012. The research related to human use was conducted in compliance with all the relevant national regulations and institutional policies. The approval of the local bioethics committee was not required due to the retrospective nature of the study (decision number: KNW-0022/KB/190/13). Informed consent was obtained from all individuals included in this study. All the CBCT volumes were obtained due to clinical indications and not for the aim of the study. The selection criteria included the following: age over 18 years and presence of all maxillary and mandibular incisors, canines, premolars and at least the first molars in the dental arches. The exclusion criteria included the following: previous or current orthodontic treatment, prosthetic crowns of mandibular incisors and canines, pathological lesions (such as inflammatory periapical lesions, supernumerary teeth, tumours and cysts), foreign bodies or previous surgery of the anterior mandible and inferior technical quality of the scans (incorrect exposure settings, low image resolution, motion artefacts and incomplete coverage of the anterior mandible).

CBCT scans of 39 males and 61 females aged from 18 to 71 years (mean age 41.34 years, 43.95 years in males and 39.67 years in females) were included in the study. A GENDEX GXCB-500 CBCT machine was applied, and the exposure parameters were the following: 120 kV, 5 mA, exposure time 6–8 s and voxel size 0.3 mm. A cylindrical field of view measuring 8 cm × 8 cm comprised upper and lower dental arches.

**Figure 1:**
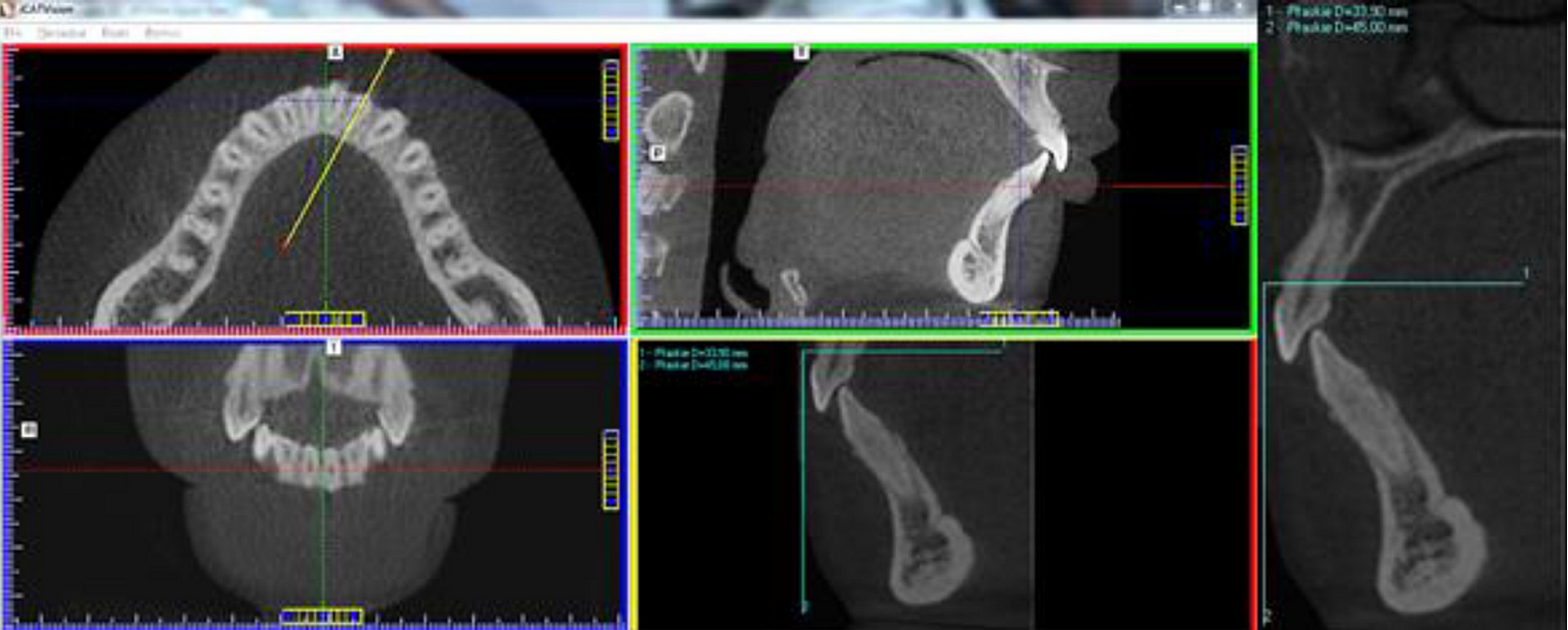
A drawing of the line determining the plane of a cross-sectional slice in the area of the examined tooth as well as a drawing of the calibration lines.

**Figure 2:**
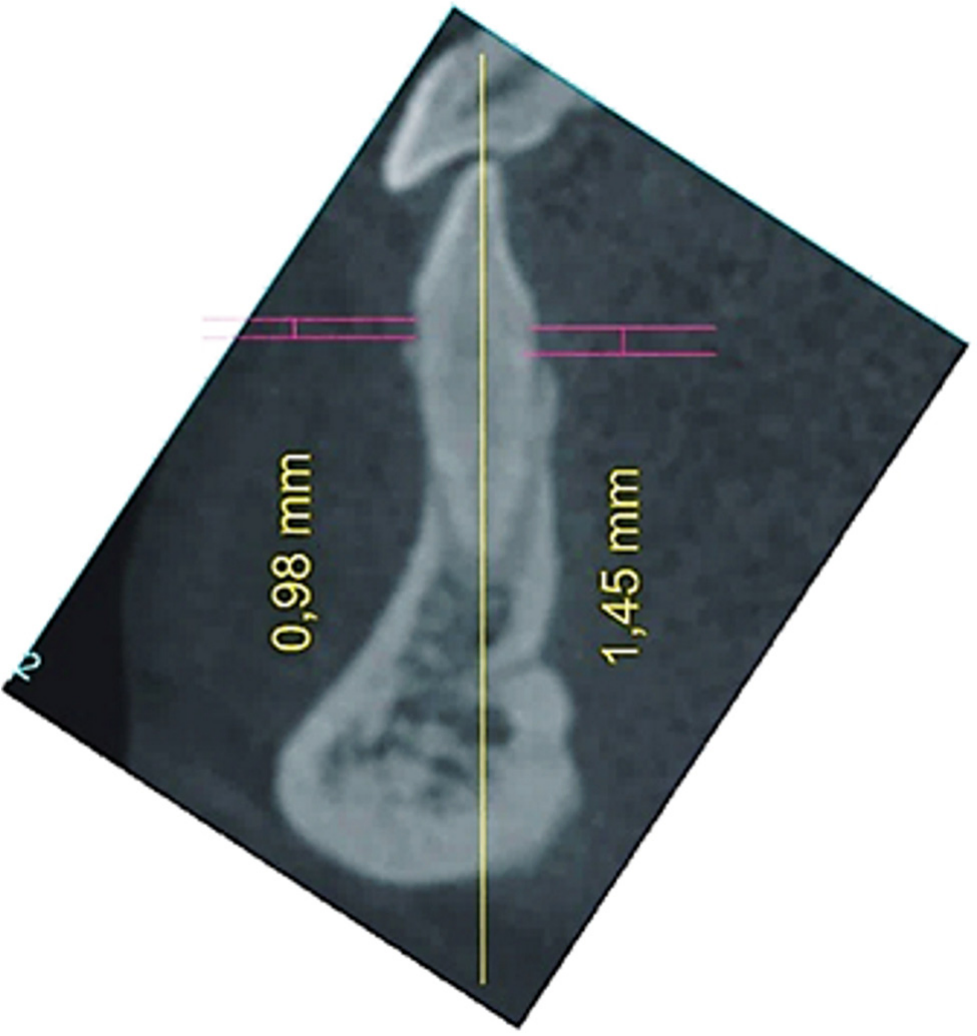
Screenshot of the CorelDraw 9 software with measurements CEJ–BBC and CEJ–LBC.

Areas of mandibular incisors and canines were selected for the analysis. First, the axial cross-section at the level of dental crevices of the mandibular anterior teeth was generated in the iCat Vision software. Next a line was drawn at the maximum convexity of the vestibular outline of the tooth and the second at the maximum convexity of the lingual surface. The lines were always drawn in the middle of the root canal cross-section (Figure 1). Two perpendicular calibrating lines of known length were drawn using IrfanView software (by Irfan Skiljan) in order to ensure measurement accuracy (Figure 1). As it was not feasible to carry out all planned measurements using the dedicated iCAT Vision software, selected images were transferred by means of the IrfanView software to the CorelDraw 9 software (serial number DX9XR – 6840J50620). Additionally, the tooth axis running through the incisal edge or cusp and the root apex was drawn. The image was then rotated making the dental axis parallel to the *Y* axis. Next, the distances between the CEJ and buccal bone crest (BBC) as well as between the CEJ and lingual bone crest (LBC) were measured (Figure 2). Every measurement was recorded three times for three consecutive days by the same observer (ASW), and the mean value was calculated.

For the purpose of statistical analysis, the patients were subdivided into two age groups. There were 70 CBCT scans of 45 females and 25 males in the age group from 18 to 49 years and 30 CBCT scans in the age group from 50 to 71 years, comprising 16 females and 14 males. Division into two age groups below and over 50 years was due to the fact that changes in bone metabolism are closely related to the age of the patient. In adult patients, the relative balance of bone metabolism is maintained. After 50 years of age, acceleration of bone loss is associated with aging and hormonal changes, more strongly in women (menopause) but also in men (andropause).

The statistical analysis was performed using Statistica for Windows software version 10 (demo version). Descriptive statistical methods, Student’s *t*-test and ANOVA were used. The significance level was set at *α* = 0.05.

## 3 Results

Detailed results of the measurements of the CEJ–BBC distance are found in Table 1. The mean distance between the BBC and CEJ was 2.32 mm ± 0.78 mm, and there were no statistically significant differences between the groups of teeth.

**Table 1:** Mean distance of CEJ–BBC and CEJ–LBC

	Mean distance between the CEJ and BBC									Mean distance between the CEJ and LBC								
Examined area	Central incisors	Lateral incisors	Canines	43	42	41	31	32	33	Central incisors	Lateral incisors	Canines	43	42	41	31	32	33
Mean (mm)	2.26	2.29	2.43	2.42	2.29	2.21	2.24	2.30	2.45	2.54	2.51	2.51	2.54	2.54	2.50	2.58	2.48	2.48
Standard error (mm)	0.08	0.08	0.09	0.09	0.09	0.10	0.08	0.09	0.11	0.10	0.09	0.09	0.09	0.09	0.11	0.11	0.11	0.10
Standard deviation (mm)	0.79	0.82	0.89	0.92	0.88	1.01	0.83	0.92	1.07	1.01	0.94	0.85	0.91	0.94	1.08	1.07	1.06	0.97
Confidence interval (95.0%)	0.16	0.16	0.18	0.18	0.17	0.20	0.16	0.18	0.21	0.20	0.19	0.17	0.18	0.19	0.22	0.21	0.21	0.19
	*p* = 0.549								*p* = 0.840									
	*p* = 0.240								*p* = 0.838									
	*p* = 0.081								*p* = 0.991									

It was proved that in males aged over 50 years, this distance was 2.84 mm ± 0.79 mm and was significantly higher than in males aged under 49 years – 2.08 mm ± 0.41 mm (*p* < 0.001) (Table 2). The same tendency was found in all groups of examined teeth (Table 3). Similar relationships were not observed in females. However, there were no statistically significant differences between the genders.

**Table 2:** Mean distance of CEJ–BBC and CEJ–LBC in different age groups of females and males

Gender	Total (CEJ–BBC)			Total (CEJ–LBC)			Females (CEJ–BBC)			Females (CEJ–LBC)			Males (CEJ–BBC)			Males (CEJ–LBC)		
Age group	All	<50 years	≥50 years	All	<50 years	≥50 years	All	<50 years	≥50 years	All	<50 years	≥50 years	All	<50 years	≥50 years	All	<50 years	≥50 years
Mean (mm)	2.32	2.19	2.62	2.52	2.26	3.12	2.29	2.24	2.42	2.45	2.35	2.71	2.35	2.08	2.84	2.63	2.10	3.28
Standard error (mm)	0.08	0.09	0.14	0.09	0.07	0.19	0.11	0.13	0.18	0.09	0.10	0.21	0.11	0.08	0.21	0.16	0.08	0.29
Standard deviation (mm)	0.78	0.76	0.77	0.85	0.60	1.04	0.85	0.90	0.72	0.73	0.67	0.85	0.67	0.41	0.79	1.01	0.41	1.08
Confidence interval (95.0%)	0.16	0.18	0.29	0.17	0.14	0.39	0.22	0.27	0.39	0.19	0.20	0.45	0.22	0.17	0.46	0.33	0.17	0.63
		0.011*			<0.001*			0.463			0.089			<0.001*			<0.001*	

Note: Values with * - statistically significantly difference.

**Table 3:** Mean distance between the CEJ and BBC in males taking into account different groups of teeth

Males	Central incisors			Lateral incisors			Canines		
Age group	All	<50 years	≥50 years	All	<50 years	≥50 years	All	<50 years	≥50 years
Mean (mm)	2.26	1.95	2.82	2.37	2.10	2.86	2.43	2.21	2.83
Standard error (mm)	0.12	0.07	0.25	0.13	0.11	0.27	0.10	0.10	0.16
Standard deviation (mm)	0.75	0.37	0.93	0.81	0.53	1.00	0.62	0.52	0.61
Confidence interval (95.0%)	0.24	0.15	0.54	0.26	0.22	0.57	0.20	0.21	0.35
		*p* < 0.001*			*p* = 0.003*			*p* = 0.002*	

The details of the distances between the CEJ and LBC for all groups of teeth are found in Table 1. The mean distance between the LBC and CEJ was 2.52 mm ± 0.85 mm, and there were no significant differences between all examined groups of teeth. However, it was found than in males aged over 50 years, this mean distance equalled 3.28 mm ± 1.08 mm and was significantly higher than in the age group up to 49 years of age, 2.10 mm ± 0.41 mm (*p* < 0.001) (Table 2). This difference was perceivable in all groups of the examined teeth (Table 4). These relationships were not found in females. There were no statistically significant differences between males and females.

**Table 4:** Mean distance between the CEJ and LBC in males in all groups of examined teeth

Males	Central incisors			Lateral incisors			Canines		
Age group	All	<50 years	≥50 years	All	<50 years	≥50 years	All	<50 years	≥50 years
Mean (mm)	2.64	2.05	3.58	2.58	1.98	3.64	2.60	2.20	3.33
Standard error (mm)	0.18	0.08	0.29	0.17	0.09	0.25	0.15	0.13	0.27
Standard deviation (mm)	1.11	0.39	1.08	1.04	0.45	0.93	0.96	0.64	1.02
Confidence interval (95.0%)	0.36	0.16	0.63	0.34	0.19	0.54	0.26	0.26	0.59
		*p* < 0.001*			*p* < 0.001*			*p* < 0.001*	

**Figure 3:**
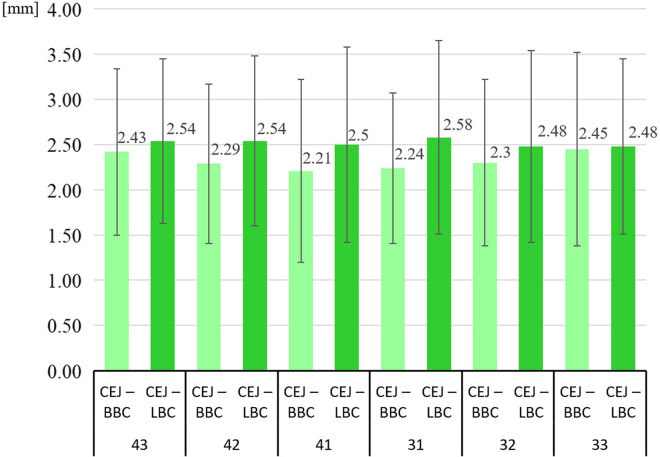
Distances of CEJ–BBC and CEJ–LBC in individual locations.

Figure 3 demonstrates a graphical comparison of the distances between the CEJ and LBC as well as BBC. Still, statistical analysis did not demonstrate any significant relationship.

## 4 Discussion

The thin gingival biotype is a risk factor of gingival recessions and increase of distance between the CEJ and alveolar ridge. This biotype is often accompanied by decrease in width of the maxillary and mandibular alveolar cortex, which is prone to resorption e.g. due to periodontal bone disease as well as during course of orthodontic treatment [22]. According to Mandelaris et al. [15]. dentoalveolar bone phenotype can be divided into two types: thick when is ≥1 mm of facial bone width and thin when is <1 mm.

The initial position of the inferior incisors and the morphology of the supporting bone are crucial for planning orthodontic treatment as tooth displacement is limited by the surrounding bone. It was proved that dental arch expansion with inclination or forward displacement of anterior teeth leads to bone remodelling. If the layer of bone is too thin, further displacement of teeth may cause fenestrations, dehiscences and predisposes to gingival recessions [6,31]. However, it was not explained whether during lingual inclination of teeth during orthodontic treatment the buccal cortex width increased. Some authors point out to such a possibility [18], while others doubt it and even report a decrease in the width of both vestibular and lingual bone during and directly after the retraction of maxillary and mandibular incisors [24,25]. The majority of authors agree that a thin vestibular cortex predisposes to gingival recessions. They do not necessarily occur immediately after the appearance of dehiscences, especially when there are no other contributing factors such as inflammation. In such cases, it is suggested to proceed with careful diagnostics and if indicated, to carry out surgical procedures aiming at increasing gingival biotype width before the onset of orthodontic treatment [3,32]. It was proved that the thin gingival biotype with gingival tissue width less than 1.5 mm correlated with lower width of the mandibular vestibular cortex [5,22]. Although the procedures of gingival augmentation do not directly influence the dimensions of the alveolar bone, they have a protective influence against the occurrence of gingival recessions. Such procedures are therefore indicated when thickness of bone covering dental roots is low, dehiscences or fenestrations are present and when the thickness of keratinized gingiva is less than 3 mm, if orthodontic treatment plan includes a considerable inclination of inferior incisors and when gingival recessions had already appeared [14]. Amid et al. [1] concluded that two gingival biotypes had significally different facial bone thickness (BT).

Until now a detailed evaluation of alveolar bone morphology of anterior mandible was challenging. Nowadays, CBCT provides precise analysis of the maxillofacial region including the maxillary and mandibular alveolar bone. CBCT provides a precise demonstration of anatomical structures [26]. Many authors proved the usefulness of CBCT in the detection and evaluation of bone defects such as dehiscences and fenestrations [13,17,20]. The sensitivity and specificity of CBCT in the diagnostics of fenestrations were estimated to be 90%, while in the assessment of dehiscences, specificity reached 95%, and the sensitivity was low and equalled only 40%. It means that many CBCT results are false negative. It can be assumed that if a dehiscence is visible in CBCT, it most probably exists, but if it is not detected, one cannot be sure that it is actually absent [6,13].

Precision of measurements in CBCT was an aim of many research studies. Kobayashi et al. [11] reported that it equalled 0.22 mm ± 0.5 mm for a voxel size of 0.125 mm, and according to Mischkowski et al. [16], it was 0.26 mm ± 0.18 mm, while for Timock et al. [28], it was 0.30 mm ± 0.27 mm. Leung et al. [13] obtained the value of 0.6 mm ± 0.8 mm at 0.38 mm voxel size.

Appropriate reference points must be used in order to correctly carry out the measurements in the CBCT images. Usually, it is easy to find a point where two tissues characterized by different densities adjoin, such as high density enamel and less mineralised cementum. In such cases, the precision of determination of measurement point depends on the voxel size.

It is often much more difficult to find a measurement point on the boundaries of two tissues of similar densities like in the case of the alveolar ridge cortex. According to Leung et al. [13] the estimated precision of determination of the location of the CEJ was 0.4 mm ± 0.3 mm and that of the buccal bone cortex, 0.6 mm ± 0.8 mm. Physical image resolution, defined as the smallest distance allowing the differentiation of two parallel lines or points as separate ones, influences the results of measurements and is one of its limitations. A precision of 0.6 mm means that areas with a thickness smaller than 0.6 mm will be depicted as areas with no bone, especially when two tissues similar in densities are taken into account. Smaller thickness of bone will result in an image with no bone at all, which is a source of errors in image interpretation and in practice in overdiagnosis of the lack of bone.

In the present study, it was proved that in 5% of evaluated cases, the level of BBC was at a distance of more than 4 mm from the CEJ, and this condition was more prevalent in females aged 50 years and more. In the studied group, this phenomenon accounted for 18.75% of cases. An increase in the distance between the CEJ and BBC is related to patient’s age as well as numerous local and general conditions leading to lowering of the alveolar bone level. In females in the peri- and postmenopausal period, the reduction of bone tissue is further intensified by unfavourable influence of hormonal changes due to the decrease in blood oestrogen levels [8].

The results of measurements of the distances between the BBC and CEJ at maxillary incisors and canines were presented by Lee et al. [12]. Their results were as follow: 2.03 mm ± 0.61 mm for central incisors, 2.46 mm ± 0.65 mm for lateral incisors and 2.71 mm ± 0.65 mm for canines. According to Januário et al. [9], the distance between the CEJ and BBC ranged from 1.6 to 3 mm. Vera et al. [29] estimated this distance to be 2.79 mm for maxillary anterior teeth. El Nahass et al. [4] obtained the following results: 2.10 mm± 0.85 mm for maxillary central incisors and 2.09 mm ± 0.72 mm for lateral incisors. Ghassemian et al. [7] found the mean values between 2.66 mm and 2.94 mm. Chan et al. [2] demonstrated that mean crestal bone level measured by means of CBCT was 2.51 ± 0.82 while for anterior teeth was 2.72 ± 0.77. In a study by Wang et al. [30] the mean CEJ–BBC distance for the maxillary central incisors was 1.8 mm ± 0.7 mm, for the lateral incisors 1.9 mm ± 0.6 mm and 2.2 mm ± 0.8 mm for the canines. They also confirmed that this distance increased with age [30]. A similar conclusion was drawn by Zekry et al. [33], who determined the range of CEJ–BBC values to be between 0.4 and 4 mm. In the present study, it was proved that the distance between the CEJ and BBC was larger in males aged over 50 years, both on the lingual and vestibular sides of the teeth. This observation was also confirmed by El Nahass et al. [4] and Ghassemian et al. [7]. The results are in concordance with the findings of many other authors who analysed the influence of age on the biology of periodontium [27]. This piece of information is crucial in the case of implant placement – their necks should be at least 2.5–3 mm below the level of the CEJ of the adjacent teeth or even deeper – depending on the applied implant system [21].

## 5 Conclusions

1. CBCT allows the determination of the location of the CEJ with high precision in relationship to the BBC and LBC.
2. In the group of males aged over 50 years, the distance between the CEJ and the bone crest differs, both on buccal and lingual sides, and this difference is statistically significant.
3. The obtained data are crucial for planning orthodontic treatment and implant placement.
